# Cell Surface Biosynthesis and Remodeling Pathways in Mycobacteria Reveal New Drug Targets

**DOI:** 10.3389/fcimb.2020.603382

**Published:** 2020-11-12

**Authors:** Moagi Shaku, Christopher Ealand, Bavesh D. Kana

**Affiliations:** National Health Laboratory Service, Department of Science and Technology/National Research Foundation Centre of Excellence for Biomedical TB Research, Faculty of Health Sciences, School of Pathology, University of the Witwatersrand, Johannesburg, South Africa

**Keywords:** tuberculosis drugs, cell surface, peptidoglycan, arabinogalactan, mycolic acids

## Abstract

*Mycobacterium tuberculosis* (*Mtb*), the causative agent of tuberculosis (TB), remains the leading cause of death from an infectious bacterium and is responsible for 1.8 million deaths annually. The emergence of drug resistance, together with the need for a shorter more effective regimen, has prompted the drive to identify novel therapeutics with the bacterial cell surface emerging as a tractable area for drug development. *Mtb* assembles a unique, waxy, and complex cell envelope comprised of the mycolyl-arabinogalactan-peptidoglycan complex and an outer capsule like layer, which are collectively essential for growth and pathogenicity while serving as an inherent barrier against antibiotics. A detailed understanding of the biosynthetic pathways required to assemble the polymers that comprise the cell surface will enable the identification of novel drug targets as these structures provide a diversity of biochemical reactions that can be targeted. Herein, we provide an overview of recently described mycobacterial cell wall targeting compounds, novel drug combinations and their modes of action. We anticipate that this summary will enable prioritization of the best pathways to target and triage of the most promising molecules to progress for clinical assessment.

## Introduction

*Mycobacterium tuberculosis* (*Mtb*), the causative agent of tuberculosis (TB), has infected approximately a third of the global population and is currently one of the leading causes of death from an infectious disease (WHO, 2019a). The emergence and global spread of drug resistant TB (DR-TB) has hampered effective control of the disease, creating an urgent need to identify novel drug targets or to re-purpose existing antibiotics (Gautam et al., 2011; Conradie et al., 2020). Considering this, cutting-edge molecular tools such as TnSeq, CRISPRi, and high-throughput whole-cell phenotypic screening with large compound libraries coupled with whole-genome sequencing are currently being used to rapidly identify drug targets and to discover novel anti-mycobacterial drugs (Grzelak et al., 2019; Rock, 2019; De Wet et al., 2020). Recently, several promising novel compounds and targets have been identified, particularly in the biosynthetic pathways of the mycobacterial cell wall (CW) (Dulberger et al., 2019; Maitra et al., 2019). Herein, we specifically review recent developments identifying new targets and inhibitory molecules ranging from those that specifically inhibit the activity of a particular enzyme in CW biosynthesis to those that may indirectly enhance the activity of certain compounds by functionally weakening the cell wall (Jeon et al., 2017; Maitra et al., 2019). Several biochemical inhibitors of the targets discussed are not approved drugs but despite this, further characterization enable a clearer understanding of essential pathways which aid in drug discovery. We focus our review on recently discovered chemical matter with proven inhibitory properties against mycobacterial CW biosynthesis enzymes and highlight some that have entered the TB drug development pipeline. We do not extensively review the biosynthesis of all CW components nor we do we provide a historical narrative of current CW targeting drugs. For this and related information, we direct the reader to several prior reviews for further information (Bhat et al., 2017; Abrahams and Besra, 2018; Maitra et al., 2019; Vilchèze, 2020). For peptidoglycan (PG), we highlight the compounds targeting the periplasmic component of polymer biosynthesis/crosslinking specifically, the MraY/MurX translocase and amidation modifications together with drugs that can potentiate the activity of β-lactams. We also summarize WhiB4-expression and its association with augmentin sensitivity. In addition, we discuss targets associated with regulation of PG biosynthesis such as the serine/threonine protein kinases and also discuss FtsZ inhibitors. For arabinogalactan (AG), we discuss inhibitors of RmlC and GlmU inhibitors as the inhibition of DprE1 by BTZ043 is widely discussed in literature.

### The Mycobacterial Cell Wall

**Figure 1:**
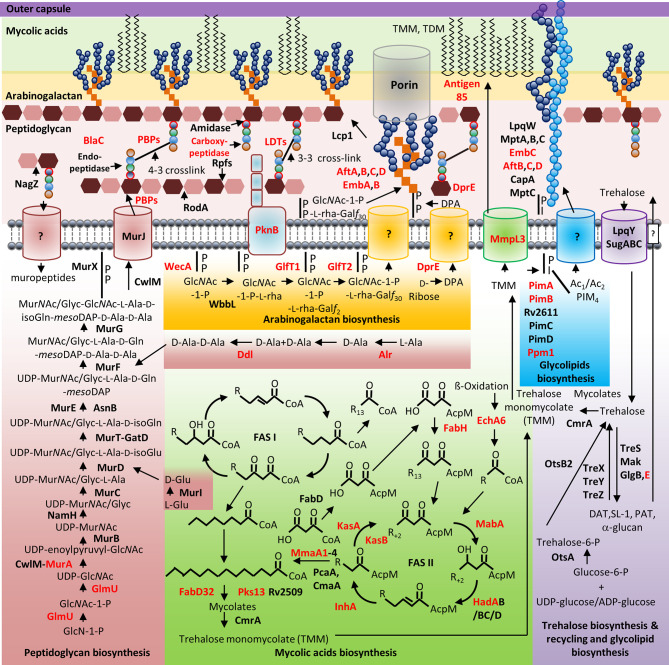
Mycobacterial cell wall and its validated and potential drug targets. Shown are the cytoplasmic and periplasmic biosynthetic pathways for the different polymers in the mycobacterial cell wall (peptidoglycan, arabinogalactan, mycolic acids, and glycolipids). Cytoplasmic and periplasmic enzymes already validated as drug targets and potential drug targets are shown in red text. Membrane channels involved in PG recycling, Glc*N*Ac-1-P-L-rha-Gal*f*30-, DPA-, Ac1/Ac2PIM4-, and surface glycolipid translocation remain to be identified (depicted by “?”).

Mycobacterial species possess a CW with biochemically diverse components, including primarily three distinct layers, namely: PG, AG, and mycolic acids (MAs) which are surrounded by a capsule (Abrahams and Besra, 2018). The capsule is comprised of proteins, polysaccharides and lipids [phosphatidyl-myo-inositol mannosides, diacyl trehaloses, phthiocerol dimycocerosates (PDIMs), and phosphatidylethanolamine] (Abrahams and Besra, 2018). In addition to these components, there are several solvent-extractable lipids including non-covalently linked glycophospholipids and inert waxes, which are well known to serve as a permeability barrier against antibiotics and play critical role in pathogenesis and survival of *Mtb*. The diverse pathways for biosynthesis of CW precursors, and subsequent processes required for transport and polymerization have been exploited for development of anti-mycobacterial drugs (Vilchèze, 2020). A select set of recently identified CW targeting compounds and those currently in clinical trials are summarized in **Figure 1** and **Table 1**.

**Table 1:** Drugs targeting mycobacterial cell wall biosynthesis pathways.

Drug target	Drug	Effect	Tested *in silico, in vitro*, *in vivo* (i.e., in animal models) or in clinical studies?	Ref
GlmU	4-Aminoquinazolines (compounds HMP-05 and HMP-15)	Inhibit PG precursor biosynthesis	*in vitro*	(Patel et al., 2020)
MurA	Fosfomycin	Inhibit PG precursor biosynthesis	N/A (*Mtb* MurA is naturally resistant to fosfomycin)	(Bensen et al., 2012)
MurB	4-Thiazolidinones	Inhibit PG precursor biosynthesis	*in vitro*	(Kouidmi et al., 2014)
PBPs	Moenomycin, β-lactams, Carbapenems and Cephalosporins	Inhibit PG cross-linking	Clinical studies (except moenomycin)	(Story-Roller and Lamichhane, 2018)
Alr	Thiadiazolidinones and D-cycloserine	Inhibits PG precursor biosynthesis	Thiadiazolidinones (*in vitro*), D-cycloserine (clinical studies)	(Kim et al., 2003; Prosser and De Carvalho, 2013; De Chiara et al., 2020)
DdlA	D-cycloserine	Inhibits PG precursor biosynthesis	Clinical studies	(Prosser and De Carvalho, 2013; De Chiara et al., 2020)
MurG	Ramoplanin	Inhibits PG precursor biosynthesis	Not tested against *Mtb*	(Lo et al., 2000)
BlaC	Clavulanate, diazabicyclooctanes (nacubactam and zidebactam), avibactam, tazobactam, and sulbactam	Inhibit BlaC β-lactamase activity	Clinical studies	(Story-Roller and Lamichhane, 2018)
LD-transpeptidase	Carbapenems and cephalosporins	Inhibit PG cross-linking	Clinical studies	(Gold et al., 2016; Lopez Quezada et al., 2020)
PknB	5-Substituted pyrimidine analogs	Inhibit PknB signaling	*in vitro*	(Carette et al., 2018)
Carboxypeptidase	Meropenem	Inhibits PG remodeling	*in vivo*	(Kumar et al., 2012)
MurX/MraY	Tunicamycin and muraymycin D2 (MD2), capuramycin, capuramycin analogs (compound UT-01320), compound SQ 641, compound X-J99620886, muramycin, caprazamycin, and liposidomycin	Inhibit PG precursor biosynthesis	Tunicamycin (*in vitro*), MD2 (*in silico*), capuramycin and capuramycin analogs (*in vitro*), compound SQ 641, compound X-J99620886, muramycin, caprazamycin, and liposidomycin (*in vitro*).	(Siricilla et al., 2015; Huszár et al., 2017)
WecA	Tunicamycin, caprazamycin, compound X-J99620886, and compound CPZEN-45	Inhibit Arabinogalactan precursor biosynthesis	*in vitro*	(Huszár et al., 2017)
GlfT1 and GlfT2	UDP-Galf derivatives	Inhibit arabinogalactan precursor biosynthesis	*in vitro*	(Abrahams and Besra, 2018)
DprE1	>15 (compounds are listed in Degiacomi et al., 2020)	Inhibit arabinogalactan precursor biosynthesis	BTZ043, Macozinone, TBA-7371 (clinical studies)	(Degiacomi et al., 2020)
AftA,B,C,D	DPA analogs	Inhibit periplasmic arabinogalactan biosynthesis	*in vitro*	(Abrahams and Besra, 2018)
EmbA,B,C	Ethambutol (EMB)	Inhibit periplasmic arabinogalactan biosynthesis	Clinical studies	(Zhang et al., 2020)
FabH	Thiolactomycin analogs	Inhibit mycolic acid biosynthesis	*in vitro*	(Senior et al., 2003)
MabA	Anthranilic acid analogs	Inhibit mycolic acid biosynthesis	*in vitro*	(Faion et al., 2020)
HadA	Thiacetazone and thiocarlide	Inhibit mycolic acid biosynthesis	Clinical studies	(Grzegorzewicz et al., 2015)
InhA	Isoniazid (INH), ethionamide (ETH), triclosan, diazaborines (compound AN12855 and AN12541), 2-(o-tolyloxy)-5-hexylpnenols (compound PT70), 4-hydroxy-2-pyridines (compounds NITD-916 and NITD-113), pyridomycin, and compound GSK693	Inhibit mycolic acid biosynthesis	INH and ETH (Clinical studies), Triclosan, diazaborines, 2-(o-tolyloxy)-5-hexylpnenols, 4-hydroxy-2-pyridines (*in vitro*), pyridomycin (*in vitro*), and GSK693 (*in vivo*)	(Dessen et al., 1995; Holas et al., 2015; Martínez-Hoyos et al., 2016)
KasA, KasB	Cerulenin, plastensimycin, TLM (thiolactomycin), and compound GSK3011724A	Inhibit mycolic acid biosynthesis	*in vitro*	(Abrahams et al., 2016; Abrahams and Besra, 2018)
FabD32	Diarylcoumarin	Inhibits mycolic acid biosynthesis	*in vivo*	(Stanley et al., 2013)
MmaA1	Compound 3-(2-morpholinoacetamido)-N-(1,4-dihydro-4-oxoquinazolin-6-yl)benzamide	Inhibits mycolic acid biosynthesis	*in vitro*	(Veeravarapu et al., 2020)
Pks13	Benzofurans (TAM16), coumestans, thiophene compounds, and β-lactones (EZ120)	Inhibit mycolic acid biosynthesis	TAM16 (*in vivo*), coumestans, thiophenes, and EZ120 (*in vitro*)	(Wilson et al., 2013; Lehmann et al., 2018; Zhang et al., 2018)
EchA6	THPPs [tetrahydropyrazo (1,5-a)pyrimidine-3-carboxamides]	Inhibit mycolic acid biosynthesis	*in vivo*	(Cox et al., 2016)
MmpL3	Compound SQ109 (a 1,2-ethylenediamine), compound C215 (a benzimidazole derivative), compounds NITD-304 and NITD-349 (indole-2-carboxamides), compound TBL-140 (a diphenylether-modified adamantyl 1,2-diamine), THPPs [tetrahydropyrazo (1,5-a)pyrimidine-3-carboxamides], SPIRO analogues [N-benzyl-6’,7’-dihydrospiro(piperidine-4,4’-thieno{3,2-c}pyran) analogues], compound E11 (an acetamide analogue), compound AU1235 (an adamantyl urea), compound BM212 (a 1,5-diarylpyrrole derivative), compound HC2091 [a N-(2-{4-chlorophenyl}ethyl)-4-thiophen-2-yloxane-4-carboxamide], and compound PIPD1 (a piperidinol-containing molecule)	Block translocation of mycolic acids to the periplasm and inhibit periplasmic mycolic acid biosynthesis	SQ109 (clinical studies), C215 (*in vitro*), NITD-304, NITD-349 (*in vitro*), TBL-140 (*in vitro*), THPPs and SPIROs (*in vivo*), Compound E11 (*in vitro*), compound AU1235 (*in vitro*), compound BM212 (*in vitro*), compound HC2091 (*in vitro*), compound PIPD1 (*in vitro*)	(Xu et al., 2017; Dupont et al., 2019; Vilchèze, 2020)
Antigen 85	Compound I3-AG85, cyclipostins, and cyclophostin analogs.	Inhibit mycolic acid biosythesis	*in vitro*	(Warrier et al., 2012; Viljoen et al., 2018)
Ppm1	Amphomycin	Inhibits glycolipid biosynthesis	*in vitro*	(Kremer et al., 2002)
TreS	α-Glycoside analogues	Inhibit surface glycolipid biosynthesis	*in vitro*	(Zhang et al., 2011; Thanna and Sucheck, 2016)
GlgE	Maltose mimics: maltose-C-phosphonate (MCP) 13, 2-deoxy-2-fluoro-α-maltosyl fluoride and deoxy-2-2-difluoro-α-maltosyl fluoride	Inhibit surface glycolipid biosynthesis	*in vitro*	(Zhang et al., 2011; Syson et al., 2014; Thanna and Sucheck, 2016)

#### Peptidoglycan Biosynthesis and Potential Targets

PG precursor (lipid II) biosynthesis includes major cytoplasmic enzymes (MurA–F) that produce UDP-Mur*N*Ac-penta-peptide or UDP-Mur*N*Glyc-penta-peptide. In Gram-negative bacteria, fosfomycin targets MurA (Bensen et al., 2012) however, as the mycobacterial MurA homologue lacks a critical active site cysteine residue required for fosfomycin binding (De Smet et al., 1999). Fosfomycin mimics able to bind the *Mtb* MurA active site remain to be designed and tested. Interestingly, unlike in other bacteria, where MurA activity is regulated by the binding of UDP-Mur*N*Ac, mycobacterial MurA activity is regulated by interaction with phosphorylated CwlM, a catalytically inactive PG amidase (Boutte et al., 2016). Several FDA-approved drugs and known MurB inhibitors, such as 4-thiazolidinones, were screened against the *Mtb* MurB homologue using docking simulations and target- or inhibitor-based approaches. As there are significant similarities in the structures of MurC and MurD/MurE and MurF, it should be possible to design inhibitors that can inhibit the Mur enzymes simultaneously (Kouidmi et al., 2014). Still within the cytoplasm, alanine racemase (Alr) catalyzes the conversion of L-alanine to D-alanine which is required to synthesize the peptide component of Lipid II (De Chiara et al., 2020). There are currently several commercial antibiotics targeting Alr (see **Table 1**), including D-cycloserine (Kim et al., 2003). D-Cycloserine has been used to treat pulmonary and extra-pulmonary TB, including MDR-TB, but this is hampered by severe toxic side-effects (Azam et al., 2016). Recently, it was shown that Alr activity remains detectable in *Mtb* exposed to clinically relevant D-cycloserine concentrations (Prosser and De Carvalho, 2013; De Chiara et al., 2020). This is due to reversible binding *via* D-cycloserine-adduct hydrolysis thus enabling dissociation and structural rearrangement within the active enzyme to regain activity. This mechanistic insight now provides a route for discovery of improved Alr inhibitors (De Chiara et al., 2020). D-cycloserine also inhibits D-Ala:D-Ala ligase (DdlA), another essential enzyme in PG biosynthesis (Prosser and De Carvalho, 2013) and improvements on the activity of this compound may yield increased activity to DdlA also.

The next step involves the translocase MraY/MurX which links the UDP-Mur*N*Ac/Glyc-L-Ala-D-Glu-*meso*-DAP-D-Ala-D-Ala to a decaprenyl phosphate (C_50_-P) to form lipid I. This enzyme has also emerged as an attractive target as it is essential in *Mtb* (Hering et al., 2018). While the natural nucleoside inhibitors of MraY, tunicamycin, and muraymycin D2 (MD2), have been available, promising efforts to design new inhibitors are emerging (Tanino et al., 2011; Chen et al., 2016; Chung et al., 2016; Mashalidis and Lee, 2020).

MurG facilitates the transfer of Glc*N*Ac from UDP-Glc*N*Ac to Mur*N*Ac or Mur*N*Glyc of Lipid I to generate Lipid II (Laddomada et al., 2019). MurG function is inhibited by ramoplanin, a lipoglycodepsipeptide which binds lipid I (Lo et al., 2000). Following this, the MurT-GatD complex and AsnB amidate the α-carboxyl group of D-glutamate and the D-carboxyl group of *meso*-DAP to form amidated Lipid II, respectively (Münch et al., 2012; Levefaudes et al., 2015). These amidation modifications are essential for PG cross-linking (Pidgeon et al., 2019) and as such, the MurT-GatD complex and AsnB remain high priority targets for development of PG targeting antibiotics.

Translocation of Lipid II into the periplasm is facilitated by MurJ, which has emerged as a possible target of newly discovered antibiotics including humimycins in gram-positive bacteria. Humimycins are potent β-lactam potentiators and display broad spectrum activity (Chu et al., 2018). However, MurJ in *Mtb* remains to be fully characterized and further work in this regard will guide the design of *Mtb* MurJ inhibitors. Ramoplanin, teixobactin, malacidin, and nisin bind periplasmic Lipid II while the glycopeptides vancomycin and teicoplanin bind the D-Ala-D-Ala terminus of lipid II preventing lipid II polymerization (Pazos and Peters, 2019). Once lipid II is translocated into the periplasm, the transglycosylase activities of PBPs and SEDS proteins facilitate the linking of the disaccharide component of Lipid II to the existing PG glycan chains (Kieser and Rubin, 2014). The transpeptidase activities of high molecular weight PBPs facilitate the formation of 4-3 cross-links between *meso*-DAP and D-Ala of adjacent penta-peptide chains. In mycobacteria, remodeling of 4-3 to 3-3 cross-links occurs *via* the co-ordinated actions of PG endopeptidases and LD-transpeptidases. The 3-3 cross-link allows for a localized increase in tensile strength of the cell wall at sub-cellular regions where there is an insufficient level of 4-3 cross linking necessary to maintain a wildtype morphology (Baranowski et al., 2018).

The moenomycin class of antibiotics inhibit transglycosylase activity of PBPs while β-lactam antibiotics inhibit the transpeptidase activity of PBPs (Ostash et al., 2010; Story-Roller and Lamichhane, 2018). Despite the successful treatment of many bacterial infections, conventional β-lactams are generally ineffective against mycobacterial species due to a chromosomally encoded β-lactamase—BlaC, which rapidly hydrolyzes the β-lactam ring (Mishra et al., 2017; Tooke et al., 2019). Consistent with this, the carbapenem class of β-lactam antibiotics are poor substrates for BlaC and when combined with a β-lactamase inhibitor, prove to be very effective in killing mycobacteria (Hugonnet et al., 2009). As a result of the early bactericidal activity (EBA) of intravenously administered meropenem plus clavulanic acid combined with oral amoxicillin (Mero/Clv/Amx) for treatment of DR-TB, the World Health Organization has recently endorsed the use of this regimen as an additional drug combination for DR-TB (WHO, 2019b). Furthermore, it was shown that carbapenems and novel cephalosporins inhibit *M. abscessus* growth (Kumar et al., 2017) and also kill non-replicating *Mtb* (Gold et al., 2016). However, the use of other carbapenems like Ertapenem in combination with Amx/Clv did not display significant EBA when compared with Mero/Clv/Amx (De Jager et al., 2020). Therefore, the search for orally bioavailable carbapenems continues.

The 2-aminoimidazoles (2-AIs) have recently been shown to potentiate the activity of β-lactams by decreasing *Mtb* protein secretion and also by increasing the CW permeability (Jeon et al., 2017). This was due to inhibition of the electron transport chain which resulted in impairment of protein secretion systems and MA biosynthesis (Jeon et al., 2019). Further investigations to identify novel β-lactamase inhibitors and the impact of novel β-lactam:β-lactamase inhibitor combinations for the treatment of DR-TB have therefore become an active research area (Story-Roller and Lamichhane, 2018).

Due to the potential of including β-lactamase inhibitors in the clinical setting, the mechanistic aspects of how *Mtb* responds to β-lactam:β-lactamase combinations, e.g., augmentin (Amx/Clv) was recently elucidated (Mishra et al., 2017). WhiB4, a cytoplasmic redox sensor, appears to co-ordinate the activity of BlaC in a redox-dependent manner. Disruption of WhiB4 increased tolerance to augmentin whereas overexpression potentiated augmentin activity against *Mtb*. Therefore, compounds that can induce increased expression of WhiB4 could enhance the bactericidal activity of augmentin against *Mtb*, this approach should be actively investigated.

#### Regulation of Peptidoglycan Biosynthesis and Potential Targets

*Mtb* expresses 11 serine/threonine protein kinases designated PknA to L, which regulate various metabolic pathways *via* protein phosphorylation. PknB and PknG are essential for intracellular survival of *Mtb* (Bellinzoni et al., 2019). PknB regulates PG biosynthesis by localizing at sites of new synthesis and activating/inhibiting PG-associated enzymes (Mir et al., 2011; Turapov et al., 2018; Kaur et al., 2019). Several kinase inhibitors that target PknA and PknB have been developed and are bactericidal for *Mtb* (Carette et al., 2018).

FtsZ, a tubulin homolog, is essential for bacterial cell division and remains an attractive target for novel antibiotics (Mathew et al., 2016). Dihydroquinolines have also been shown to possess inhibitory activity against mycobacterial FtsZ (Carro, 2019). Loss of LamA, another mycobacterial cytoskeletal factor, influence polar growth dynamics (a phenotype associated with heterogeneity in susceptibility to antibiotics), indirectly enhancing susceptibility to rifampicin (RIF) and cell wall targeting drugs (Rego et al., 2017). Similarly, loss of FtsX, a regulator of cell division, enhanced susceptibility to RIF (Mavrici et al., 2014). A recent whole-cell screening approach identified compound APYS1, an aminopyrimidine-sulfonamide, with potent activity indirectly inhibiting *Mtb* Wag31/DivIVA, an actin-like protein required for cell wall biosynthesis and cell elongation (Singh et al., 2017).

#### Arabinogalactan Biosynthesis and Potential Targets

AG biosynthesis is initiated in the cytoplasm by WecA, resulting in a “linker” region that serves as the site of attachment to PG (Alderwick et al., 2015; Huszár et al., 2017). WecA is a target of tunicamycins and caprazamycin derivatives (Huszár et al., 2017). The next steps yield units that are polymerized in the periplasm by AftA-D and EmbA-B (targets of ethambutol), see **Figure 1**. The AG complex is then ligated to PG by Lcp1, which has also recently emerged as an attractive target in the *Mtb* CW (Hett et al., 2008; Harrison et al., 2016). The crystal structure of a LCP homologue in *Staphylococcus aureus* has been solved and this provides insight into structure-guided design of inhibitors of this enzyme family (Li et al., 2020). For further insight, the reader is directed to a recent review that comprehensively describes the biosynthesis of galactan in *Mtb*, with a focus on drug discovery (Konyariková et al., 2020).

Another promising target, DprE1, acts as a flavoenzyme which uses the cofactor FAD (flavin adenine dinucleotide) to oxidize DPR (decaprenyl-phospho-ribose) to a keto-intermediate. This is then reduced to decaprenyl-phospho-arabinose (DPA—a substrate for AG biosynthesis) by DprE2 using NADH (Brecik et al., 2015). DprE1 localizes to the CW, negating the need of some DprE1 targeting drugs to enter into the cytoplasm (Brecik et al., 2015). DprE1 was first discovered as the target for benzothiazinones (BTZs), since then more than 15 DprE1 inhibitory compounds some of which have entered clinical trials have been identified (Degiacomi et al., 2020). The most promising BTZ compound to date, BTZ043, displayed a MIC of 1 ng/ml (0.23 nM) and is currently progressing through clinical trials. Macozinone (MCZ, also known as PBTZ169) is a BTZ043 derivative which has been medicinally optimized and has entered clinical trials. TBA-7371 is another DprE1 inhibitor with high potency and has also entered clinical trials (Degiacomi et al., 2020).

#### Mycolic Acid Biosynthesis and Potential Drug Targets

Targeting MA biosynthetic enzymes holds significant potential for developing new anti-tubercular drugs as MAs influence permeability and sensitivity to hydrophobic antibiotics (Jankute et al., 2015). Enzymes in the FAS-I and FAS-II pathways synthesize and facilitate the correct folding of short and long-chain fatty acids, respectively. Several enzymes in the FAS-I and FAS-II pathways are essential for viability and have been targeted, see **Figure 1** and **Table 1** (Sassetti et al., 2003; Baran et al., 2020). InhA is the target of isoniazid (INH) and structural analogues such as ethionamide (ETH) activated by the catalase-peroxidase KatG (Dessen et al., 1995). Novel InhA inhibitors (indicated in **Table 1**), unlike INH and ETH, do not require prior activation and have potential for treatment of DR-TB while some are also bactericidal against non-replicating *Mtb* (Holas et al., 2015; Martínez-Hoyos et al., 2016; Flint et al., 2020). KasA and KasB are targets of cerulenin, plastensimycin, thiolactomycin, and indazole sulfonamides (Abrahams et al., 2016; Abrahams and Besra, 2018). Tetrahydropyrazo (1,5-a)pyrimidine-3-carboxamides (THPPs) have recently been shown to target EchA6, a catalytically inactive enoyl-CoA hydratase required for shuttling fatty acyl-CoA esters into FAS-II for MA biosynthesis (Cox et al., 2016). MmaA1 is a target of the compound 3-(2-morpholinoacetamido)-*N*-(1,4-dihydro-4-oxoquinazolin-6-yl)benzamide (Veeravarapu et al., 2020). Benzofurans, coumestans, thiophenes, β-lactones target Pks13 (Wilson et al., 2013). Diarylcoumarins inhibit FabD32 and possess high bactericidal activity against *Mtb* (Stanley et al., 2013).

The mature MAs are translocated to the CW *via* the membrane embedded transporter—MmpL3 (Grzegorzewicz et al., 2012; Xu et al., 2017). Before translocation, the MAs are attached to trehalose by mycolyl-transferases to form trehalose monomycolates (TMMs) (Jankute et al., 2015). TMMs are attached to AG by the mycolyltransferase Ag85 complex. TMMs may also be converted to trehalose dimycolates (TDMs) by the Ag85 complex before being attached to AG (Jankute et al., 2015). Ag85C is a target of the compound I3-AG85 causing inhibition of incorporation of MAs into the mycobacterial CW (Warrier et al., 2012). Cyclipostins and cyclophostin analogs have been shown to inhibit Ag85 enzymes (Viljoen et al., 2018). MmpL3 is currently one of the most promising anti-TB targets (Vilchèze, 2020). Several diverse compounds (see **Table 1**) display inhibitory activity against MmpL3 either by direct binding or disruption of membrane potential (Xu et al., 2017; Dupont et al., 2019; Vilchèze, 2020; Yang et al., 2020).

#### The Variance of Cell Wall Targets in Clinical Strains

*Mtb* clinical isolates display differences in the composition of the CW that should be noted for drug discovery approaches (Moopanar and Mvubu, 2020). Specific differences in abundance of pthiotriol dimycerosate and PDIM, have been noted in clinical isolates of the East Asian/Beijing, Indo-Oceanic, and Euro-American lineages (Krishnan et al., 2011). These lipids form a hydrophobic barrier to antibiotics and therefore the differences in lipid profiles or differences in the abundance of enzymes associated them can be correlated with differential susceptibility to antibiotics. For example, the proteomic profile of an INH resistant clinical strain of the Euro-American lineage (T-family), and an INH resistant lab strain were analyzed by LC-MS/MS and this revealed an altered abundance of FAS-II pathway enzymes required for MA biosynthesis. This altered abundance of FAS-II enzymes was compensated for through alternative enzymes including FabG4, HtdX, FadD13, and members of the *mymA* operon, which has been shown to play a role in MA biosynthesis (Singh et al., 2005; Mehaffy et al., 2018). Although the change in protein abundance only affected the abundance of the two more abundant MAs in the clinical INH resistant strain, these lineage specific differences in the abundance of CW associated biosynthetic enzymes should be considered during development of new anti-mycobacterial drugs. A later study conducted by Seepe *et al*. in two drug susceptible and resistant East-Asian (Beijing) clinical isolates exposed to INH further elucidated the differences in MA composition amongst clinical isolates and found differential expression of FAS-II pathway enzymes highlighting the importance of considering the basis of lineage specific differences for CW targeted TB drug development (Seepe, 2011).

Despite the promise of using β-lactam:β-lactamase combinations, it should be noted that susceptibility to this combination treatment is variable among clinical isolates (Gonzalo and Drobniewski, 2013; Forsman et al., 2015; Zhang et al., 2016). A recent study assessing 89 clinical isolates from South Africa demonstrated that approximately half of the clinical isolates studied were hyper-susceptible to Amx/Clv as compared to the reference strains (Cohen et al., 2016). Whole genome sequencing of Amx/Clv hypersusceptible LAM4 isolates identified polymorphisms in the genes *aftD*, PE-PGRS genes, *pks12*, and *ubiA* associated with CW biosynthesis. Hence, screens for new PG targeting compounds should include different lineages of *Mtb*, together with representatives of drug resistant strains.

TnSeq has recently been used to conduct fitness profiling of clinical isolates, identifying notable differences in the requirement of various genes for viability and antibiotic susceptibility (Carey et al., 2018). Several CW biosynthesis genes including *murI*, *pbpA*, *ldtB*, *otsA*, *pimE*, *pssA*, *papA3*, and CW associated lipoprotein biosynthesis genes (*lppL* and *lppX*) were found to be differentially required for fitness in various clinical isolates (Carey et al., 2018). Moreover, TnSeq was also used recently to identify genetic variants that influence drug efficacy *in vivo*. Several *Mtb* mutants displayed altered susceptibility to TB drugs in the murine model of TB disease, many of which were associated with CW pathways described herein. The findings suggest that genetic variants that may be associated with drug resistance in clinical *Mtb* isolates do not alter *in vitro* drug susceptibility but can still influence drug efficacy *in vivo* (Bellerose et al., 2020). This suggested that the genetic backgrounds of different clinical strains can impact the applicability of new anti-mycobacterial drugs in the clinical setting.

## Future Directions and Concluding Remarks

A significant amount of work has been done in the identification of potential drug targets in the CW and their inhibitory compounds, however, only a few lead compounds have entered clinical trials (https://www.newtbdrugs.org/pipeline/clinical). Improved strategies to test and fast-track the development of these compounds including the use of TnSeq and CRISPRi in clinical strains to study target vulnerability is of importance given the high prevalence of DR-TB. When considering these recent developments, the mycobacterial cell surface continues to hold promise for the development of shorter, more effect TB treatments.

## Author Contributions

MS and CE wrote the article. MS prepared the figure. BK provided the concept, critical review, and edited the final draft. All authors contributed to the article and approved the submitted version.

## Funding

This work was supported by funding from the South African National Research Foundation (to BDK and CE), the South African Medical Research Council (to BK, CE, and MS) and the Centre for Aids Prevention Research in South Africa (CAPRISA, to BK).

## Conflict of Interest

The authors declare that the research was conducted in the absence of any commercial or financial relationships that could be construed as a potential conflict of interest.
